# Can Hepatocellular Carcinoma be Cured by Microwave Coagulation?

**DOI:** 10.1155/1998/37589

**Published:** 1998-08

**Authors:** C. H. Scudamore

**Affiliations:** University of British Columbia 910 West 10th Avenue Vancouver V5Z 4E3 Canada


terminal pancreatic portion of the cyst 12 years after excision [4]. Complete cyst excision can be safely performed in nearly all cases and is now well established as the treatment of choice for choledochal cysts. A good case can be made to extend total excision to include the intrapancreatic portion of the cyst without fear of pancreatic duct damage. The entire cyst is removed thereby eliminating the chance of later malignancy.

### *References*

[1] Todani, Y. *et al.* (1995). Biliary Complications after Excisional Procedure for Choledochal Cyst. *J. Pediatr. Surg.*, **30**, 478 – 481.

[2] Ohi, R. *et al.* (1990). Surgical Treatment of Congenital Dilatation of the Bile Duct with Special Reference to Late Complications after Total Excisional Operation. *J. Pediatr. Surg.*, **25**, 613 – 617.

[3] Miyano, T. *et al.* (1996). Hepaticoenterostomy after Excision of Choledochal Cyst in Children: A 30 year Experience with 180 cases. *J. Pediatr Surg.*, **31**, 1417–1421.

[4] Yoshikawa, K., Yoshida, K. and Shirai, Y. *et al.* (1986). A case of carcinoma arising in the intrapancreatic terminal choledochus 12 years after primary excision of a giant choledochal cyst. *Am. J. Gastroenterol*, **81**, 378 – 384.

Alastair Millar
Sid Cywes
Department Paediatric Surgery
Institute of Child Health
Red Cross Children's Hospital
Rondebosch 7700
South Africa

### Can Hepatocellular Carcinoma be Cured by Microwave Coagulation?

### ABSTRACT

*Sato, M., Watanabe, Y., Ueda, S., Iseki, S., Abe, Y., Sato, N., Kimura, S., Okubo, K. and Onji, M. (1996) Microwave coagulation therapy for hepatocellular carcinoma. Gastroenterology; 110, 1507 – 1514.*

*Background and Aims:* **Surgical resection is not always feasible in patients with hepatocellular carcinoma. Microwave coagulation therapy has been used as an alternative to resection, and its efficacy has been evaluated.**
*Methods:* **Nineteen patients with unresectable hepatocellular carcinoma underwent microwave coagulation therapy through laparotomy (n=12), laparoscopy (n=5), or thoracotomy (n=2) because of advanced liver cirrhosis and/or intrahepatic metastases. One nodule was treated in 13 patients, and two to five nodules were treated in 6 patients; tumor size ranged from 5 to 90 mm. Patient outcomes were studied.**
*Results:* **Microwave coagulation therapy created a reproducible regional necrosis. Fourteen patients underwent potentially curative treatment; the remaining 5 patients underwent palliative treatment (n = 4) or incomplete tumor coagulation (n = 1). Of the 31 nodules treated, 28 underwent complete tumor ablation. Only 2 patients undergoing laparoscopic microwave coagulation therapy developed local recurrence. The coagulated area subsequently shrank. Patients showed rapid recovery without hepatic dysfunction. Thirteen patients, including 2 long-term survivors, are alive either without tumor (n=10; 14 –64 months) or with tumor (n = 3; 17 – 22 months). Six patients died of hepatocellular carcinoma (n=4) or liver insufficiency (n=2).**
*Conclusions*: **This preliminary study suggests the efficacy of microwave coagulation therapy, including safety and potential curability, in patients with hepatocellular carcinoma with advanced liver cirrhosis and multifocal or central tumors.**

*Keywords:* Hepatocellular carcinoma, microwave coagulation, acetic acid injection

### PAPER DISCUSSION

Hyperthermia has been employed clinically as one of a variety of multi-modal therapies for malignancies. There is increasing evidence to support the hypothesis that cancer cells are more sensitive to heat than normal cells possibly due to alterations in blood supply in neoplastic tissue and a decreased ability for neovascular beds to vasodilate. Regional and systemic hyperthermic therapies have many technical and physiological disadvantages over local treatment.

Cautery or coagulative necrosis is produced by a variety of modalities and has long been used as a means of destroying malignant tissues in situ. Modern electrical use of alternating current for coagulation was popularized by Harvey Cushing and W.T. Bovie in 1928. However, coagulation (charring of tissues) tends to limit the heat distribution and size of destruction by increasing tissue impedance. If tissue temperature is kept below 100°C, conductive heating spreads over a larger area.

The recent interest in radiofrequency (RF) ablation (high frequency alternating current) stems from two concepts. One, tumors can be destroyed in situ without the traditional resection for cure, thus potentially reducing the morbidity and mortality associated with major liver resections. Two, small tumors if destroyed, have a curative potential and if cure is not possible, there may be a palliative benefit in reducing tumor volume.

Radiofrequency ablation combines the two concepts of cell death via heating the tissues to lethal temperatures (>70°C for 5 min) and coagulation necrosis in the center of the lesion and the needle tract, reducing bleeding from the tract. Current RF probes are small diameter (15 French) and some probes have multiple retractable hooks each of which contains an active electrode and a thermistor to monitor tissue temperature, and provide a predictable spherical zone of tissue destruction.

Cryotherapy ablation functions in a similar fashion but the technology is more expensive, cumbersome, and the treatment times tend to be significantly longer. Alcohol injection has been successful in treating small hepatocellular carcinomas, but its use is limited in larger primary tumors and desmoplastic metastases, where the tissue permeability with alcohol is unreliable. As in cryoablation, RF has the disadvantage of the heat sink where high volume blood flow removes heat, which may protect malignant cells adjacent to major blood vessels such as hepatic veins. This potential problem may be reduced with the addition of alcohol injection through the hollow RF needle.

Sato, *et al.*, have shown RF destroys tissues several centimetres in diameter using an open technique and several applications. This technique may be effective in patients who would normally not be resection candidates. However, the RF technology may provide its greatest advantage in treating smaller tumors, for shorter durations using a percutaneous or laparoscopic approach. These patients can be treated as out patients thus maximizing the potential for debulking tumors, reducing medical costs, and potentially preventing a major surgical procedure with significant impacts on the patient's well being.

RF is a new technology which has great potential in adding to the already expanding field of tumor ablation. With greater world experience, RF's place will become clear.

### *References*

[1] Cushing, H. (1928). Electro-surgery as an aid to the removal of intracranial tumors. *Surgery, Gynecology and Obstetrics* **47**, 751–84.

[2] Strom, F. ed. (1983). Hyperthermia in cancer therapy. Boston: G. K. Hall Medical Publishers, 1–376.

[3] Haines, D. and Watson, D. (1989). Tissue heating during radiofrequency catheter ablation: a thermodynamic model and observations in isolated perfused and super perfused canine right ventricular free wall. *PACE*, **12**, 962–76.

[4] Buscarini, L., Rossi, S. and Fornari, F. *et al.* (1995). Laparoscopic ablation of liver adenoma by radiofrequency electrocautery. *Gastrointestinal Endoscopy*, **41**, 68–70.

[5] Rossi, S., Di Stassi, M. and Buscarini, E. (1995). Percutaneous radiofrequency interstitial thermal ablation in the treatment of small hepatocellular carcinoma. *Cancer Journal of Scientific American*, **1**, 73–81.

[6] D'Agostino, H. and Solinas, A. (1995). Percutaneous ablation therapy for hepatocellular carcinomas. AJR, **164**, 1165–7.

C. H. Scudamore,
Associate Professor Surgery
University of British Columbia
910 West 10th Avenue
Vancouver V5Z 4E3, Canada

